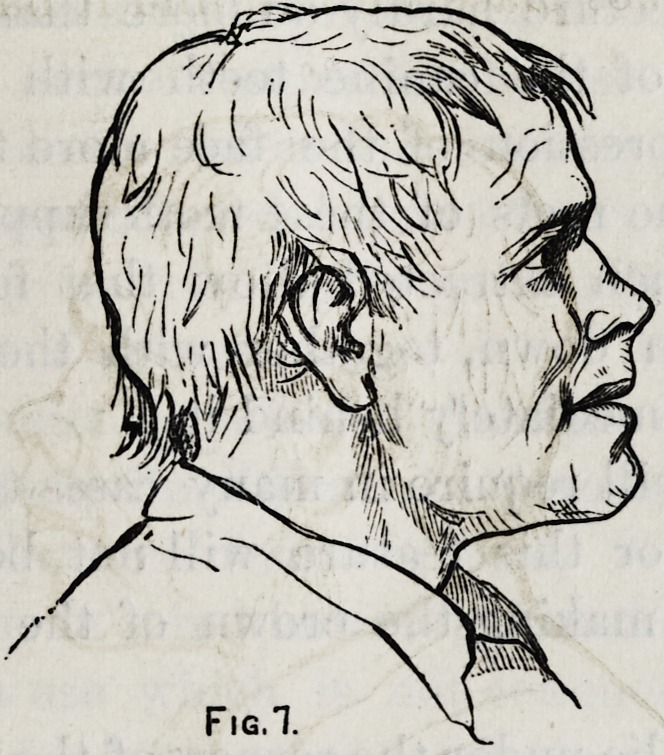# Dentistry as a Fine Art

**Published:** 1868-08

**Authors:** Norman W. Kingsley

**Affiliations:** Prof. of Dental Art and Mechanism, New York College of Dentistry.


THE
### AMERICAN JOURNAL
OF
### DENTAL SCIENCE.

Vol. 11. THIRD SERIES--AUGUST, 1868. No. 4.

### ARTICLE I.

### *Dentistry as a Fine Art.*

By NORMAN W. KINGSLEY, Prof. of Dental Art and Mechanism, New York College of Dentistry.

CONCLUDING ARTICLE.†

GENTLEMEN:—In my previous lectures upon this subject, I have addressed you as Art Students; Members of the same family and co-workers with sculptors and painters.

I now address you, not simply as belonging to the great brotherhood of Artists, but as specialists, who are to bear in many respects far greater responsibilities than either sculptors or painters.

In your hands are to be placed living forms to be manipulated; either for better or worse—forms which are the most sacred of all the works of the Creator, the same form chosen by the Great First Cause when he manifested himself to the sons of men, and the only form in which we shall ever see him.

With this sacred treasure, marred by accident and disease you will be constantly familiar, and to enable you to suc-

† I feel that an apology is due to those readers of the Journal who have taken any interest in my preceeding articles for my delay in concluding the series. The laborious duties of a lengthened session in the college were followed in the spring by an illness which has prevented the earlier preparation of this article, and now I think the substance will prove quite as profitable to the reader to present it in the form of a lecture, substantially as it was delivered before the class for their instruction. N. W. K.

cessfully hide its deformities has been my endeavor in educating you as artists.

In presenting the general principles which should govern you in this work, I may not always have made you appreciate your trust as I would wish, nevertheless when you come into actual practice if you will but remember the views which have been advanced, and endeavor faithfully to make the application, you will agree with me that it is one of the most interesting and absorbing of studies.

It is my purpose in the present lecture to make the practical application of those principles for you so far as it is possible in the absence of an opportunity for a demonstration upon a variety of living faces.

Following me therefore in my imagination, we have presented to us a patient with countenance deformed by the entire loss of teeth, superior and inferior, alveolar processes more or less absorbed—wasted and unsupported muscles—sunken cheeks and lips, and a nose whose cartilaginous portion has lost its hereditary character.

No fancy picture in reality but a portrait drawn from the every day experience of the dentist.

Toward the rebuilding of this ruin you have already made considerable progress.

Under the instructions in the mechanics of dentistry, you have learned to accurately adapt plates of some suitable material to the gums of both upper and lower jaws and these being placed in position, now for the first time your *artistic* labors commence.

Provided therefore with props to be placed in the mouth to determine the length of the face, also with some plastic material, of which nothing is better than common bees-wax, and some simple instrument with which to manipulate your wax and you have all the accessories for the exercise of the highest order of artistic talent.

If I were to model out of inanimate clay, a head either ideal or from nature my thought and my first works would partake of very much the same character as yours will with this patient now before you.

We will therefore work together—you at your living pa tient and I at my ideal.

First then let us establish the profile.

This is not only primary in the order of your work but it is of primary importance. This is the central point around which all your other modeling revolves.

This becomes the standard which governs all the other features. The profile well chosen, all the other features will be made to harmonise with it, and according to the profile will correspond, in form, the beauty of all the other features. No face was ever repulsive where the profile was beautiful, and no face can be made beautiful where the profile is ugly. Of all importance is it then, that at this point we carefully study our plans for restoration.

We shall the better do this by having some standard of beauty in our minds as an ideal toward which we are working. We must also bear in mind that it will only be possible for us to approximate to a very limited extent, our ideal. Nevertheless if we will intelligently set ourselves to ascertain the elements of beauty in our ideal—what contributes to this beauty and what detracts from it, we shall have fixed in our minds general principles, which we can apply to a greater or less extent to every work.

To illustrate this point I have here a drawing of the head of the Apollo Belvedere.

Fig. 1.

This statue takes its name as you know from the Belvedere gallery in which it stands, in the Vatican palace at Rome, and has been an accepted standard of male beauty for hundreds of years. I do not present this drawing for our analysis solely upon the approval of others.

My own impressions of the statue when I first became acquainted with it, were, considering its high reputation rather those of disappointment; but as I have brought a more extended observation in my comparison of it with other statues and with living faces, I must concede that in no other masculine face have I found so much beauty.

So careful have I been to bring you a correct drawing that I have made this by a simple mechanical process from a cast in my possesion of the full size and it is therefore absolutely correct. The object of the artist in the representation was to portray the highest type of physical, rather than intellectual beauty and the character of this god gave him abundant opportunity.

The general line of the forehead and nose you will observe is the same. In many of the Greek statues it is a single and straight or nearly straight line from the tip of the nose to the top of the forehead and it is this line that forms the distinctive characteristic of the Grecian profile.

But that portion to which I call your special attention is the nose, mouth and chin.

None of these features will admit of any material modification without detracting from the beauty.

Fuseli, a celebrated lecturer on art, said, shorten the nose of the Apollo by but the tenth of an inch and the god is destroyed. Observe therefore the relation that the nose bears to the upper lip, also the relation of the upper to the lower. The nostrils take the same general direction of the mouth—were they to be raised at their posterior boundary it would give to the face a sneering and contemptuous look or were they drawn down, it would give a surly and morose expression.

You will observe not only the gentle curve of the upper lip in its outline but also its comparative shortness and its prominence as related to the lower.

You will also note the fulness and curve of the lower lip but particularly the decided depression midway between the mouth and the chin.

Let me sum up briefly the chief elements of beauty. A short finely curved and prominent upper lip. A full round and less prominent lower lip. A strongly marked depression at the base of lower lip giving roundness and character to the chin.

We will go farther, and give the comparative size of the features. The forehead above the eyebrows is one-third of the length of the face—the nose is one-third, and the mouth and chin are equal to one-third. Of the distance from the nose to the base of the chin, the upper lip is one fourth, the lower lip is one fourth and the chin is equal to both or one half. Thus we have made a very good analysis of this profile, and having accepted it as a standard will compare others with it.

I have here the head of another Grecian divinity—Medusa.

FIG. 2

In many respects it is the most remarkable female head I have ever seen. Make your analysis of this profile and you will see that it possesses the same general characteristics; and these characteristics of the lower part of the face are elements of beauty wherever as a whole you find them. Thus, while at the present day the pure Greek type is very rarely seen, we nevertheless do see, in all handsome profiles very much the same outline in the lower part of the face that I have indicated above. The variations bring in the upper half of the face and not in the lower.

I have here a drawing of another type of profile; one that you will recognize as not uncommon in life.

Indeed if I were to describe the American type, I should be as much inclined to give that name to this form of feature as to any other, it being quite as universal as any other type which is distinctive and possesses the elements of beauty.

FIG. 3.

We find here much the same proportion as in our standard, and also developed to a considerable extent the characteristics of beauty in the lower part of the face. I present you here another drawing of the same face, after the loss of the upper and lower teeth for some months, and you will here mark the beginning of that deformity which it is your duty to remedy.

FIG. 4.

The mouth is sunken, the lips compressed, the end of the nose flattened, the nostrils drawn down and the whole line of beauty in the lower part of the face gone.

It will be interesting to follow this developement a little farther and I have here another drawing of identically the same face in all the minutia of detail, except the region around the mouth. Here is exhibited that wonderful transformation from youth and beauty to age and ugliness ; and all those peculiarities which were noticed in the earlier stages are still more stikingly developed.

FIG 5.

In this last drawing which is the size of life,* I have raised the lower jaw so that the face is shortened only the quarter of an inch and you cannot but be impressed by the effect produced.

You have it now in your power to remodel this face and it is very important that you carefully consider whether you can make any greater improvement than simply to restore the features to their original form and position. I think the more you study it, the more certain will be your conclusion that the original form in this case, harmonizes better with the upper features, than any change it is possible for you to make.

If you had a living model now before you with these features and were to experiment upon it, you would probably find that any material variation from the original form would show a want of correspondence between the lower half of the face and the upper.

* The drawing here given is a reduction by photography of the drawing used in the lecture, which was life size.

I desire now to call your attention to one of the ugly developements of nature and one in which, when what we call deformity takes place we will find it but a step towards beauty.

FIG. b.

This face you will also recognize as a type of many that you have seen and one which at first glance seems to have hardly a redeeming feature, and yet if you will analyze it, it is only the lower half of the face that is decidedly ugly.

In fact, it is only the cartilaginous and movable part of the nose, together with the two lips, which give this beastly look. The forehead is not bad, neither is the chin. Let us consider now what we will attempt here for improvement. If we fancy that to make a mouth here like the mouth of the Apollo would give beauty to the face we should find ourselves disappointed. Such a mouth in conjunction with the other features which we cannot alter, would only be making a deformity of a beautiful individual member.

But there is no danger of your committing such an error, you can only manipulate these features to a limited extent. You *can* depress the lower end of the nose, raise the nostril, retract and shorten the lips and shorten and improve the face by raising and advancing the chin. Supposing therefore that when the entire teeth have been lost and the alveolar process absorbed, we exercise our skill. Instead of attempting to restore the features to their original position as we would in our former illustration, we should study to avoid that, and at the same time should study to avoid the appearance of a sunken mouth.

Fig. 7.

This drawing which I now show you will illustrate my meaning. The chin is here raised one quarter of an inch; in this case the advantage is decided; in Fig. 5, the same process produced deformity.

Again too it is not always by the absorption of processes and the retreating of the lips that we get improvements.

It is not uncommon to find the upper lip less prominent than the lower, and that too when the teeth are fully developed underneath. In such a case it is manifestly desirable, if the free movement of the muscles of the upper lip will permit, to advance it to the line of beauty.

I have here a number of drawings of other faces differing in a marked degree from those which I have produced but these are sufficient to illustrate the principles which I am advancing. I must here caution you particularly to study with great care the movements of the mouth.

It is very possible that you may have produced a most desirable change to be observed when the mouth is in repose, but when seen in action the expression from overstrained and unduly-taxed muscles; is horrid.

In your modelling you will therefore pay great respect to expression; for a pleasing expression is of far more consequence than a scientifically beautiful outline or contour.

This leads us very naturally to the steps to be followed subsequently to the establishment of the profile.

I can in this lecture hardly do more than allude to them. The extraction of the canine teeth with their long roots destroys the expression of the face more than that of any other teeth. The roots of these teeth support the wings of the nose, and when extracted allow that feature to be disagreeably drawn down, together with the formation of a deep wrinkle immediately behind it.

Your model will require in many cases to be carried well up at this point or this feature will not be restored. But you must *avoid* making the crown of the canine teeth too prominent.

These crowns lie under the corners of the mouth and there is hardly anything more disagreeable than to see the corner of the mouth strained when in repose, and to reveal when opened, two tiger like fangs.

Be careful also that you do not strain the upper lip so that its beautifully curved line is obliterated and the mouth represent only a comparatively straight incision.

Preserve also the groove which should indicate the median line below the nasal septum, which is a mark of beauty.

With the lower lip also use the utmost care, that only its edge be advanced and that it be entirely undisturbed at its junction with the chin and if possible, at the corner of the mouth let the lower lip fall within the upper.

We come lastly to consider the support and consequent form which you will give the cheeks.

If all the processes which I have already indicated to you have been skillfully performed, you will find this last comparatively easy of accomplishment.

With all the other features in harmony and only sunken cheeks to fill out to correspond, your labor will be light. But here too you will be liable to over-strain them. It is very easy by over-doing this, to suggest a swollen face, or the disgusting practice of a quid of tobacco in the mouth.

In a restoration of the features after the teeth have been for a long time removed, the cheeks and the lips not having been supported meantime by art; both the comfort of the patient and the necessity of preserving the indentity, would suggest that the entire restoration be not accomplished at once. In like manner when the features are to be remodeled and the muscles taxed beyond their original development, the change can be made gradually, with ease, and without sacrifice of expression.

The muscles must be allowed freedom of action and it will sometimes be quite difficult to permit this, and at the same time give the most desirable form.

It will be borne in mind however that the muscles can be developed into a use which is not common with them and certain expectations for the future may be predicated on this fact.

In all these efforts, the law of harmony must not be forgotten: a skinny forehead, angular eye-brows, hollow eyes, and depressed temples associated with full lips, plump cheeks and a well developed chin, will strike even an ordinary observer as an incongruity. In the study of human faces the student of nature will find new and pleasing wonders continually, and to carry out the law of harmony his highest power of discrimination will be in constant requisition.

He will find to his astonishment that what might be termed mechanical symmetry is lacking in every face.

So accustomed do we become to the general configuration of the human head that we rarely if ever view it critically.

A close comparison of one side with the other of almost any face will detect grave departures from uniformity.

A straight line from the centre of the forehead to the centre of the chin will not necessarily bisect the nose, showing that the median line is not a straight line but a curve. Neither the eyes nor the eye-brows will occupy the same angle to the median line, one side will be higher than the other; and the same is true of the mouth.

The distance from the corner of the mouth to the outward corner of the eye will not measure the same on both sides.

The horizontal circumference of the the skull being ovoid, the face does not occupy the precise front, it being longer from the anterior median line to the posterior median line on one side than the other.

By standing behind a person and looking over the head thus bringing the face reversed to the the eye these deviations from mechanical perfection may be the more readily observed.

Their recognition by the portrait sculptor is of consequence and of little less importance is it to the dentist. From it we may learn that often in a restoration a slight variation in the fullness of the cheeks will harmonize better with the surrounding features of that side than if both were equally plump.

In the preparation of the foregoing articles I have found it no easy task to reconcile two subjects which in the minds of most readers are so widely separated as Dentistry and the Fine Arts, and yet I am more profoundly impressed than ever with the intimate relations of the two. While the cursory reader will derive but little benefit from their perusal I am not without hope that the minds of some may be stimulated to a higher appreciation of the fine arts and especially " Dentistry as a Fine Art."

## Figures and Tables

**Fig. 1. f1:**
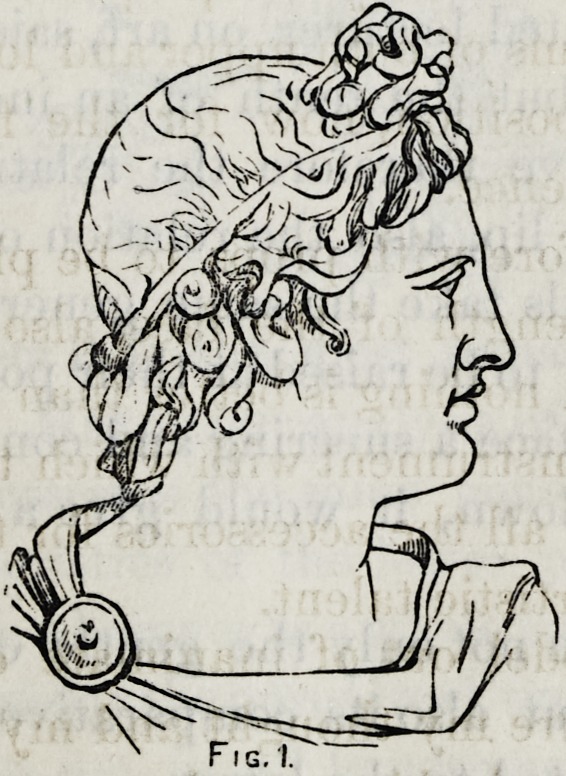


**Fig.2 f2:**
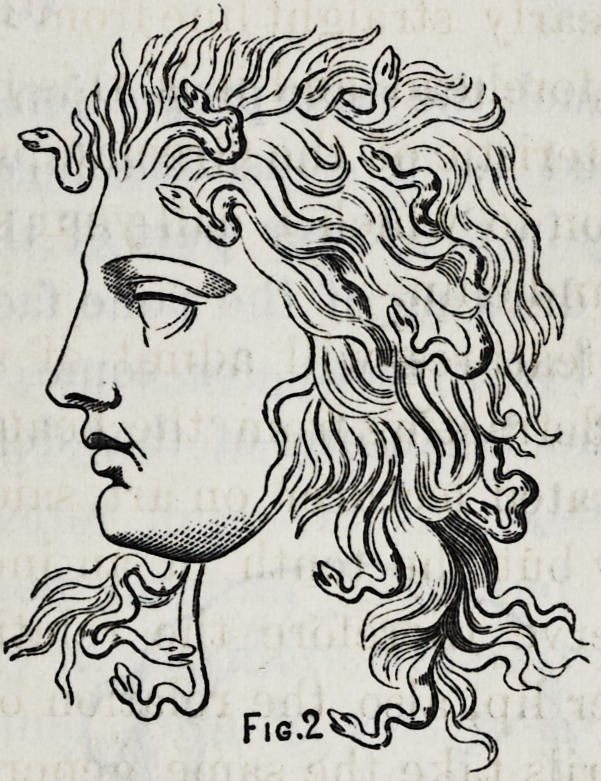


**Fig. 3. f3:**
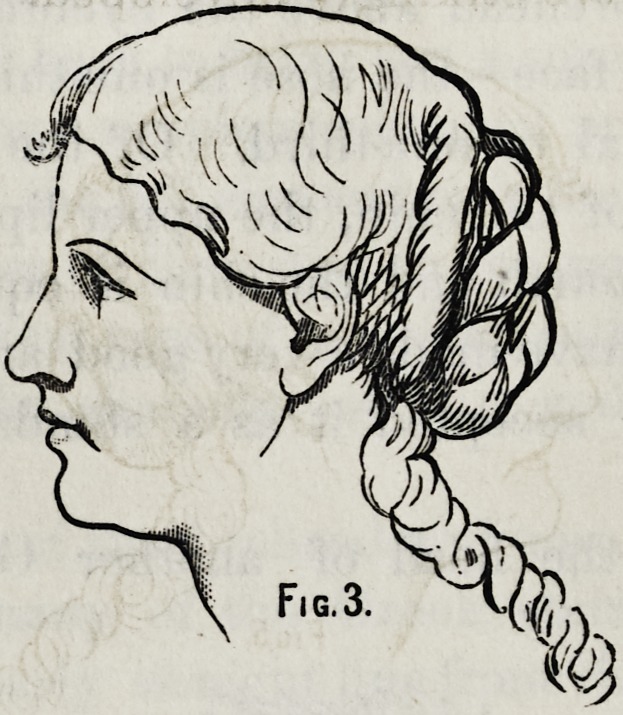


**Fig. 4. f4:**
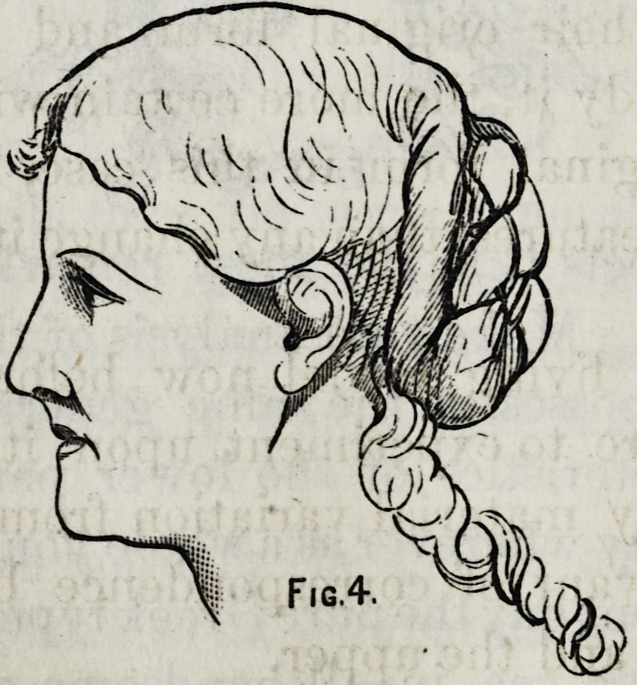


**Fig 5. f5:**
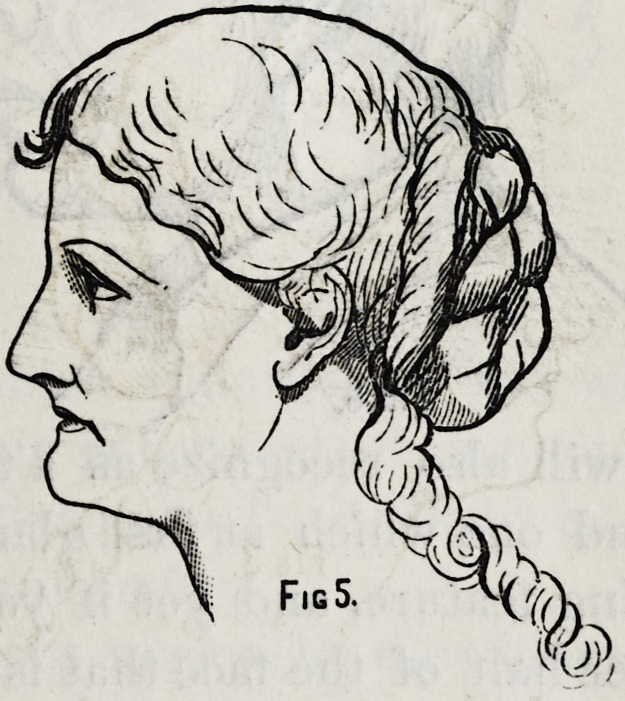


**Fig. 6. f6:**
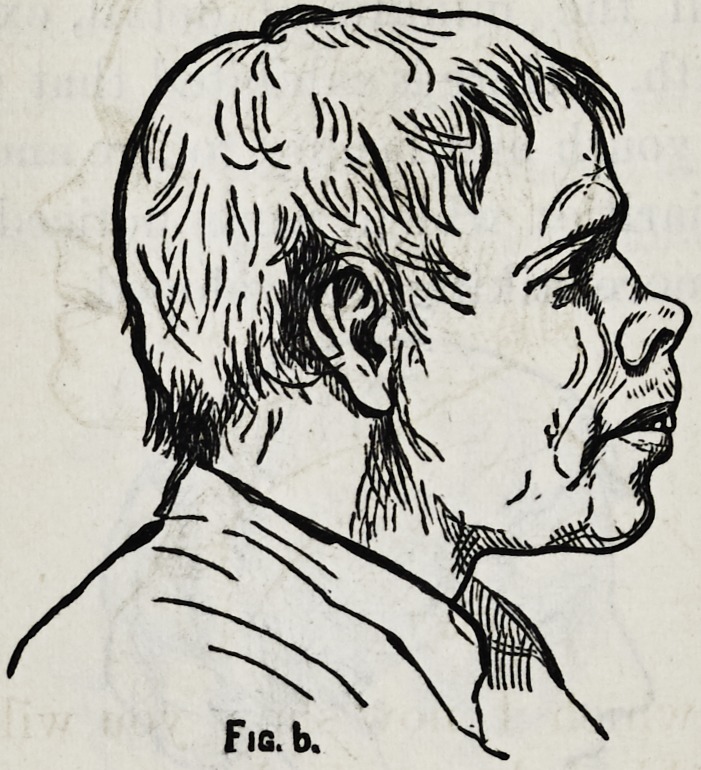


**Fig. 7. f7:**